# Allegheny County Medical Society

**Published:** 1888-12

**Authors:** 


### ALLEGHENY COUNTY MEDICAL SOCIETY.

A special meeting was held at Pittsburg, Pennsylvania., August 21, 1888, W. M. Brinton, M.D., President, in the chair.

Dr. E. T. PAINTER read a paper on "A Case of Œsophageal Stricture."

The patient, æt. about 38, complained of difficulty in swallowing food, and its regurgitation, both liquid and solid, and of inability to drink cold water or other cool drink. It was first noticed that food would not move on in response to the usual movements of deglutition, and that its ownward progress was assisted by a few gentle raps on the back. This symptom first showed itself about six years ago. From this slight difficulty in the passage of solid food to the stomach, the patient gradually found herself compelled to subsist wholly on liquid foods, and these could be retained only when taken at a certain warm temperature. Neither water at ordinary temperature, nor cool drink of any sort, nor solid food, had entered the stomach in a period of years. She was emaciated and destitute of physical vigor.

An examination of the œsophagus with a bougie proved the existence of a band, which would resist the further progress of the instrument till the constriction willed to give way, when the bougie would easily slip into the stomach. Neither had the diameter of the bougie, nor the flexibility of a tube, nor force, seemed to have anything to do with passing through the constricting ring. Passage beyond the constriction could be made only when the ring was so disposed and inclined. There had been no pain or hæmorrhage. There was no history of the introduction of a foreign body and its impaction, or of the swallowing of a strong acid or strong alkali. No aneurism was evident. There is no history of carcinoma. The constriction was sixteen inches from the lower incisors. Dysphagia and regurgitation, which prevented the patient retaining sufficient nourishing food, were the only symptoms given.

As drugs, massage, the passage of a flexible tube, and the Faradic current had failed to accomplish any good results, he resolved to give a thorough trial to the galvanic current locally applied. He employed from six to ten cells of a galvanic chloride of silver battery, placing a sponge electrode joined to the negative pole in one hand, and an œsophageal electrode connected with the positive pole within the constricting ring. This electrode consisted of an ovoid shell, seven-sixteenths of an inch by three-fourths of an inch, of perforated hard rubber, which could be unscrewed in the middle, and had sufficient space within for absorbent cotton which came in contact with a small expanse of platinum, and that in turn was united by an insulated wire to the battery.

The battery used gives a current absolutely constant in character; and a water rheostat served to differentiate the strength of the current. The applications were made three times a week for a few weeks, then twice a week, each treatment lasting from six to twelve minutes.

At the termination of the first treatment the following contingent took place. On attempting to withdraw the œsophageal electrode, it came easily in response to traction for a few inches, when it was seized by a contraction of the œsophagus, and there firmly held for a few seconds. This peculiar accident did not happen a second time. After each treatment the patient placed herself in a recumbent position for a half-hour. At the fourteenth visit the electrode was passed through and beyond the point of stricture without the knowledge of the patient, nor did she feel any sensation of opposition. At dinner, after the fifteenth application, the patient ate meat and bread and butter.

In about three months twenty-five applications were made, obtaining most decided improvement. The contracting ring persists, but its irritability has disappeared. The patient eats, without regurgitation, of what others at the table partake, restricting herself in only one item of food—meat, which is cut fine for her. She drinks water and milk freely. For a time the stomach, unaccustomed to such foreign substances as bread and butter, strawberries, cheese, etc., made the patient aware of its change in function by dyspeptic disturbances.

The interesting points in this case are these: The ease with which a diagnosis of dyspepsia could have been made, its long existence, its obstinacy under manifold treatment, the continued presence of the stricture without its irritability, and the rapid change in character of the constriction under the influence of the galvanic current.

Dr. DUFF related the history of a case of purpura simplex in a child. The case improved under treatment for several days, when the condition changed, and is described by Dr. Duff as follows:

The child was suffering very materially from its teeth, and was biting its gums and crying a great deal. I examined its gums, and found them very much swollen. I thought to relieve it by scarifying the gum; this was in the evening about five or six o'clock. I scarified the gum over one of the canine teeth and went home. About daylight I was called to the house, and found the child lying in bed, blood all over the mother's clothing, the bed bloody, and the child pulseless. I administered restoratives and worked with it for some time until it revived somewhat. In the afternoon I saw it again and the purpuric spots had reappeared on its back, and there was some hæmorrhage from the bowels as well as hæmorrhage from the bladder. The child died the next morning. There was no history of hæmorrhage in the family, with the exception of the father, who has frequently had severe hæmorrhages from the nose. There is no history of tubercular trouble in either branch of the family.

Dr. J. J. BUCHANAN presented a paper on "Fractured Patella Treated by Wiring."

The patient is a German laborer, and his fracture was the result of direct violence. He stated that the accident happened in the middle of the day of June 30. He continued to do his work till evening, but on the following day found that he was unable to stand on the limb. It is probable that the blow broke the bone, but the capsule held together till evening. When he was brought to the hospital, five days afterward, the joint was considerably distended and the fracture easily recognized, but the lower fragment seemed to be very small. The operation was on the eleventh day after the injury.

The most scrupulous precautions against sepsis were taken. Instruments and appliances were put through the same course of preparation as for laparotomy. Continuous irrigation with 1-2500 sublimate solution was employed, and the transverse incision was made to the full extent of the rent in capsule. The lower fragment was not larger than a chestnut. The capsule was much lacerated, and a number of narrow shreds hung into the joint; the joint contained a great deal of clotted blood and bloody fluid. The joint was thoroughly washed out and all loose pieces and ragged ends and edges of capsule were cut away with scissors. The fractured surfaces were refreshed by the vigorous use of a curette.

A single hole was drilled through each fragment, the drill entering about three-eights of an inch from the line of fracture and emerging at the cartilaginous border of the fractured surface.

A special meeting was held September 18, 1888, W. M. Brinton, M. D., President, in the chair.

Dr. W. P. Munn, read paper on "Chronic Intestinal Obstruction: Report of Case and Operation."

Mrs. E. A., æt. 31, the mother of five children, the youngest aged eleven months, the oldest twelve years of age. Two years ago she began to be of a constipated habit, but had no special medical attention. Early in 1887 she became pregnant, and in October was delivered at full term. Her confinement was uncomplicated, and her recovery uninterrupted. She resumed her household duties sooner than I thought advisable, on account of not being able to secure proper help. She never became quite as robust as she had been, and complained of constant dragging pain in the back and pelvis, but especially on the left side; the tendency to constipation continually increased, her general health failed in spite of tonic treatment, and her face assumed a cachectic appearance.

In January I was called to see her on account of a more than ordinarily obstinate attack of constipation, the bowels not having moved for seven days, although enemata had been employed every day. During the next three days she took, successively, calomel, 20 grains in divided doses, castor oil, sulphate of magnesia, and sulphate of soda, but without any effect. Then I prescribed an aloin, strychnia, belladonna, and ipecac tablet every three hours; by mistake she took one of these every hour until twenty had been taken, at the same time using hot-water enemata twice daily. No effect was produced beyond excessive tympanites. Digital examination of the rectum showed it to be empty; *per vaginam* a sensitive induration was discovered posterior to, and slightly to the left of, the uterus.

Dr. W. S. Foster now saw her in consultation. Placing her in Sims' position, with the hips well elevated, we introduced the long tube of a stomach pump into the rectum, until it encountered some obstacle at or near the sigmoid flexure, which was partially passed after a little careful manipulation, and then injected about a quart of hot water into the bowel. She could feel this passing through the descending and transverse colon, but on rising and using the chamber, only the water retained in the rec tum was evacuated; the remainder drained away slowly after several hours, but only one or two small lumps of fæcal matter passed. Five times in the next three days I repeated this maneuver, adding each time an ounce of castor oil to the warm water in the syringe, and she finally had a free *fluid* movement, very large in quantity. This was on the fourteenth day, and all this time, although the abdomen was somewhat distended and the pelvic pain continued as before, there was no elevation of temperature or any other sign of peritonitis.

Following this attack she improved much, but it was found necessary to keep the contents of the bowels in a fluid condition by the daily administration of salines; neglect of this for twenty-four hours was invariably followed by a recurrence of the old symptoms, and necessitated a return to the long tube of the stomach pump, as before. All this time she kept up the daily use of the hot vaginal douche; but notwithstanding this, the induration in the posterior *cul de sac* appeared to increase; the left ovary could be isolated by careful bimanual examination, and though somewhat enlarged and very tender, it was very slightly movable. The right ovary seemed to be normal in size, location, and mobility, but was abnormally sensitive. Her pelvic pain radiated from the ovaries and was constantly increasing in severity.

Early in March an attack of peritonitis threatened, but was averted by the free and persistent use of saline cathartics. Following this, she gained strength for several weeks, and at the end of the month she was able to be out of bed for a few days, but the improvement was only temporary, and the end of April found her once more bed-ridden.

Two attacks of localized peritonitis occurred between this time and the 10th of June, and each time the patient rallied more slowly. Her appetite began to fail, and even the semi-daily administration of salines failed to keep the contents of the bowels soluble. The constant catharsis was also a severe drain on a naturally strong constitution.

The exact nature of the obstruction could not be determined, but it was certainly at or near the sigmoid flexure. The possible conditions were from, *first*, simple stricture or narrowing of the bowel; *second*, a neoplasm, perhaps malignant, involving the bowel at this point; *third*, constriction by fibrinous bands, resulting from peritonitis; *fourth*, a tube or ovary, probably the left, attached to the bowel as a result of inflammatory action and forming the obstruction.

Palliative and expectant treatment and persistent use of electricity having utterly failed, and the patient's condition becoming progessively worse, operative interference seemed the only resort, with the view of removing the obstruction if possible; and if not removable, to then stitch the bowel to the abdominal wall and establish a fæcal fistula.

Accordingly, on July 13, assisted by Dr. W. S. Foster, I opened the abdomen by a linear central incision five inches in length.

The *left ovary* was cystic and about an inch and a half in diameter, dislocated into the *cul de sac* of Douglas, and adherent by its posterior surface to the last movable portion of the intestine. The uterus was enlarged and slightly retroverted. The ovarian pedicle and the Fallopian tube were ligated with twisted silk, and removed. The right ovary, on examination, proved to be cystic also, and was removed. Adherent to the great omentum there was found a colloid body about a half-inch in length, similar to those I have seen in cases of ruptured ovarian cysts with colloid contents. This was ligated and clipped off, but was unfortunately mislaid, and the opportunity for a microscopical examination lost.

Convalescence was uninterrupted after the second day, although up to that time persistent bilious vomiting occasioned some anxiety. The bowels were fully evacuated on the fourth day by the use of saline purgatives, and after that moved naturally and unaided, except by an occasional aloin, strychnia, and belladonna tablet every day or every second day. One slight attack of constipation, lasting four days, occurred six weeks after the operation. She is still in Ohio, and writes to her husband that her strength does not return rapidly, though her appetite and digestion are good.

In reply to an interrogation, Dr. John Ashhurst, Jr., has kindly given me the following statement of similar recorded cases:

PHILADELPHIA, August 17, 1888.

DEAR SIR: Your letter of 16th inst. is received. In the International Encyclopædia of Surgery, vol. vi, p. 74, I have tabulated thirty-seven cases of laparatomy for obstruction of the bowels from tumor, strictures, ulcers, etc., ten of these thirty-seven cases having ended in recovery. I have since added to my list eleven cases, of which five are said to have terminated favorably, so that the figures now stand thus:

| | |
|---|---|
| Recovered | 15 |
| Died | 30 |
| Undetermined | 3 |
| | — |
| Total | 48 |

Very truly and respectfully,
JOHN ASHHURST, JR.

